# Neurosteroids and Seizure Activity

**DOI:** 10.3389/fendo.2020.541802

**Published:** 2020-09-30

**Authors:** Barbara Miziak, Magdalena Chrościńska-Krawczyk, Stanisław J. Czuczwar

**Affiliations:** ^1^Department of Pathophysiology, Medical University of Lublin, Lublin, Poland; ^2^Department of Child Neurology, Medical University of Lublin, Lublin, Poland

**Keywords:** neurosteroids, ganaxolone, seizures, epilepsy, catamenial epilepsy

## Abstract

Still circa 25% to 30% of patients with epilepsy cannot be efficiently controlled with available antiepileptic drugs so newer pharmacological treatment options have been continuously searched for. In this context, a group of endogenous or exogenous neurosteroids allosterically positively modulating GABA-A receptors may offer a promising approach. Among endogenous neurosteroids synthesized in the brain, allopregnanolone or allotetrahydrodeoxycorticosterone have been documented to exert anticonvulsant activity in a number of experimental models of seizures—pentylenetetrazol-, bicuculline- pilocarpine-, or 6 Hz-induced convulsions in rodents. Neurosteroids can also inhibit fully kindled seizures and some of them have been reported to counteract maximal electroshock-induced convulsions. An exogenous neurosteroid, alphaxalone, significantly elevated the threshold for maximal electroconvulsions in mice but it did not potentiate the anticonvulsive action of a number of conventional antiepileptic drugs against maximal electroshock-induced seizures. Androsterone not only elevated the threshold but significantly enhanced the protective action of carbamazepine, gabapentin and phenobarbital against maximal electroshock in mice, as well. Ganaxolone (a 3beta-methylated analog of allopregnanolone) needs special consideration for two reasons. First, it performed better than conventional antiepileptic drugs, diazepam or valproate, in suppressing convulsive and lethal effects of pentylenetetrazol in pentylenetetrazol-kindled mice. Second, ganaxolone has been evaluated in the randomized, double-blind, placebo-controlled phase 2 trial in patients with intractable partial seizures, taking maximally 3 antiepileptic drugs. The initial results indicate that add-on therapy with ganaxolone resulted in reduced seizure frequency with adverse effect being mainly mild to moderate. Possibly, ganaxolone may be also considered against catamenial seizures. Some positive effects of ganaxolone as an adjuvant were also observed in children with refractory seizures and its use may also prove efficient for the management of neonatal seizures associated with hypoxic injury. Neurosteroids positively modulating GABA-A receptor complex exert anticonvulsive activity in many experimental models of seizures. Their interactions with antiepileptic drugs seem ambiguous in mice. Initial clinical data indicate that ganaxolone may provide a better seizure control in patients with drug-resistant epilepsy.

## Introduction

Epilepsy is a neurological disease which affects more than 70 million of the global population, 30% of whom are patients with refractory epilepsy (drug-resistant epilepsy) (1–3). Research conducted globally has invariably shown that, in terms of risk factors, epilepsy is hardly ever caused by a single determinant. A number of underlying causes are usually present, together with a genetic predisposition to the disease. Huge progress in terms of medical and pharmacological studies has facilitated the discovery of antiepileptic drugs (AEDs), which in one third of cases can suppress epileptic seizures, yet they do not affect in any way the root cause of the disease, so one cannot expect any improvements in the long-term prognosis of patients. Other solutions include surgical treatments, which might seem the most effective therapy. However, also in this case it has to be borne in mind that not every patient can undergo such a procedure (1, 4). In recent years other strategies in epilepsy treatment have emerged; however, there is still a great necessity for other options to be pursued, among which the most effective would be treatment impacting the etiopathogenesis of the disease, which would help to manage the condition, particularly in patients with refractory epilepsy (1, 2). Refractory seizures still affect about one third of patients with epilepsy in spite of a number of newer AEDs which appeared on the market over the last two decades (5). Consequently, there is a continuous search for more effective strategies and possibly neurosteroids could reduce the number of refractory epilepsy patients. When a newer or potential AED is evaluated, it is used in the form of an add-on therapy to the already existing antiepileptic treatment (6) and this is also the case with neurosteroids tried in patients with drug-resistant epilepsy (see below).

## Methods

Literature search for this review was generally based on English language articles with a few exceptions. PUBMED databases were the main source of relevant papers and the search areas included: neurosteroids, neurosteroids and seizure activity, neurosteroids and AEDs, neurosteroids and epilepsy. Some references of particular importance were searched for in the most relevant publications extracted from PUBMED.

## Neurosteroids

**Figure 1:**
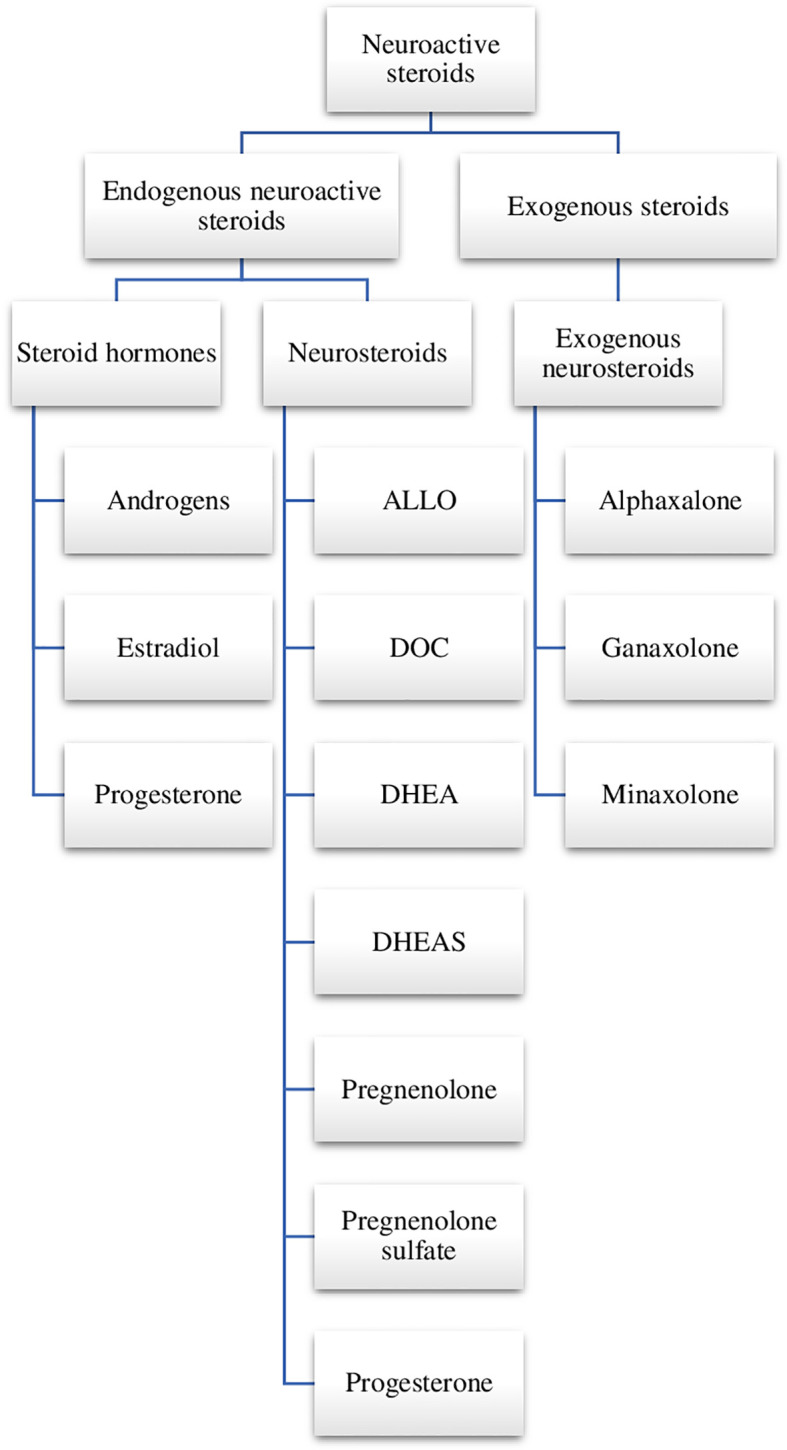
Neuroactive steroids. ALLO, allopregnanolone; DOC, deoxycorticosterone; DHEA, dehydroepiandrosterone; DHEAS, dehydroepiandrosterone sulfate; SGE-516, 1-((3R,5R,8R,9R,10S,13S,14S,17S)-3-hydroxy-3,13-dimethylhexadecahydro-1H-cyclopenta[a]phenanthren-17-yl)-2-(2H-1,2,3-triazol-2-yl)ethan-1-one.

Among the endogenous and exogenous steroid compounds, it is possible to distinguish neurosteroids—compounds possessing the ability to modulate neuronal activity and affect the physiology of the central nervous system (CNS). There is a distinction between steroid hormones which are secreted outside the nervous system (by endocrine glands) and neurosteroids (7). The term neurosteroids was introduced by Baulieu in 1981. This name is usually given to steroids which are synthesised *de novo* from cholesterol in the CNS, mainly by the glial cells and by neuronal mitochondria through pathways which are independent of the endocrine system. At first, only the elevation of dehydroepiandrosterone sulfate (DHEAS) was observed that was found in high concentrations in the brain long after gonadectomy and adrenalectomy. Only at a later stage did it occur that DHEAS could be also synthesised in the brain. Other neurosteroids were discovered with time, including androstenedione, pregnenolone (with their sulfates and lipid derivatives) as well as tetrahydrometabolites of progesterone and deoxycorticosterone (DOC) (7). There are two groups of neurosteroids—natural (endogenous—produced in the brain) and exogenous **(****Figure 1**).

Mechanism of neurosteroid action is connected with allosteric regulation of gamma-aminobutyric acid (GABA)-A receptors, *N*-methyl-d-aspartate (NMDA) receptors, alpha1 glycine receptors, sigma receptors and voltage-dependent calcium channels (8–11). Neurosteroids potentiate synaptic GABA-A receptor function and also activate δ-subunit-containing extrasynaptic GABA-A receptors that mediate tonic currents and thus may play an important role in neuronal network excitability and seizure susceptibility (12).

Depending on their concentration, neurosteroids can directly activate receptors (in the case of high concentrations) or act as potent positive allosteric agonists (in the case of low concentrations) (12–15). In addition, these compounds can have a positive or a negative impact on the modulation of GABA-A receptor activity. The mechanism based on positive modulation was observed in the case of such neurosteroids as allopregnanolone (5alpha-pregnan-3alpha-ol-20-one, ALLO), androsterone, progesterone, DOC, and tetrahydrodeoxy-corticosterone (THDOC). Through their binding to a specific place of the receptor, the above compounds bring about changes to its conformation, which results in the influx of Cl^−^ ions into a neuron. Local hyperpolarization of the neuron which occurs in such situations leads to the inhibition of the cell’s activity (12, 16). Neurosteroids in fact allosterically modulate synaptic and extrasynaptic GABA-A receptors, exhibiting a greater potency for the latter, containing delta subunits. Positive modulators, acting on extrasynaptic GABA-A receptors, located in the dentate gyrus of the hippocampus, may reduce seizure susceptibility (17). The second group includes compounds (pregnenolone sulfate and DHEAS), which suppress the GABAergic response through the mechanism of negative GABA-A receptor activity modulation. The above mechanism is based on the inhibition of chloride current, resulting in cell depolarization and activation (18). Further studies have shown that intracerebroventricular pregnenolone sulfate enhanced the convulsive activity of peripherally administered NMDA in mice and this effect was possibly dependent on the elevated concentration of hippocampal alanine, a precursor of glutamate and a probable co-agonist at the NMDA receptors (19).

The existing evidence also points to sigma1 receptor as a possible target for neurosteroids. In fact, some neurosteroids were demonstrated to displace sigma1 receptor radioligands *in vivo* and *in vitro* (20). Moreover, steroid sulfate esters (DHEAS or pregnenolone sulfate) differentially affected NMDA-induced noradrenaline release from preloaded hippocampal slices (DHEAS was an enhancer whilst pregnenolone sulfate—an inhibitor). The inhibitory effect upon this parameter was shared by the high-affinity inverse sigma agonist and the sigma antagonist, haloperidol, attenuated the effect of steroid sulfate esters (21). The authors of this study conclude that DHEAS behaves as a sigma agonist whilst pregnenolone sulfate—as a sigma inverse agonist. Interestingly, progesterone behaved identically to haloperidol so it seems to be a sigma receptor antagonist (21).

Literature data draw attention to the effect of progesterone on calcium signaling—progesterone-calcium signaling hypothesis, which may explain therapeutic benefits of neurosteroids—a reduction of inflammation and edema, prevention of demyelination or inhibition of excitotoxic neuronal death. These detrimental effects are associated with significant elevations of the intracellular calcium concentration. Consequently, neuroprotection provided by progesterone seems dependent on the direct inhibition of voltage-gated calcium channels (22).

## Neurosteroids and Seizure Activity

### Progesterone and Its Metabolite

There are studies which indicate that neurosteroids might also be important when it comes to the epileptogenesis described as the latent period. The latent period, which is a seizure-free period, has been defined as interval between the effect of the primary brain insult and the onset of the first spontaneous seizure. During this time, pathological changes occur in the brain, converting the normal brain into one that generates seizures (23).

In studies conducted on rats in the pilocarpine model of status epilepticus, it was found that during the latent period of epilepsy a higher expression of cytochrome P450 cholesterol side-chain cleavage (P450scc) enzyme was noted. This enzyme takes part in the brain synthesis of neurosteroids (24). Cholesterol is delivered by translocator protein (TSPO) into mitochondria in which P450scc is located (in the inner mitochondrial membrane). Next, cholesterol is cleaved to produce pregnenolone and is metabolized to tissue-specific steroids by enzymes, which operate in the endoplasmic reticulum (24, 25). Blocking neurosteroid synthesis by finasteride (a 5alpha-reductase and neurosteroid synthesis inhibitor) in rats after status epilepticus induced by pilocarpine, resulted in terminating the latent period and moreover, finasteride was evidently proconvulsant in rats with spontaneous seizure activity occurring after the latent period (24). Finasteride was also tested upon the anti-epileptogenic activity of progesterone in the mouse hippocampus kindling model of epileptogenesis (26). The neurosteroid synthesis inhibitor completely inhibited the progesterone anti-epileptogenic effect (reflected by the retardation of the kindled seizures) which could suggest that this particular action of progesterone may be dependent upon ALLO (26). Seyle et al. (27) were probably the first to conduct this type of study, in 1942, in which they evaluated the progesterone activity in pentylenetetrazol animal model, using immature male rats, demonstrating the hormone’s anticonvulsant properties. In the subsequent years, other researchers also confirmed these results in studies conducted on both male and female rodents, using various progesterone doses and convulsive animal models, including the amygdala kindling model (28, 29), hippocampal kindling model (30), WAG/Rij rats, the genetic absence model (in this case, an increase in the number and duration of spike-wave discharges was demonstrated) (31) or kainate model (32). Billiar et al. (33), in turn, evaluated the distribution and metabolism of progesterone, the products of its metabolism, and estradiol, but this time they administered a constant infusion of [3H]- or [14C] progesterone and estradiol [3H] to female rhesus monkeys. The experiments showed that the hormone levels were significantly higher in the cerebral tissues than in the carotid arterial blood, with the estradiol concentration being highest in the anterior pituitary (20 times). In the case of progesterone, its lowered concentrations were observed in the “*central gray (P less than 0.05); the concentration levels were the same for the amygdala, hippocampus, preoptic-anterior hypothalamus, cerebellum, hypothalamus, thalamus, and anterior pituitary; and were higher in the cervical spinal cord, optic chiasm, mesencephalon, medulla oblongata, and pons*” (33), when compared with the control group. Many years of research have shown that the administration of exogenous progesterone results in its cerebral-tissue concentration’s being tripled when compared to peripheral tissue levels. In the case of the administration of both progesterone and its metabolite—5alpha-pregnan-3,20-dione (5alpha-DHP) to rats (*iv* administration), it was demonstrated that the compounds accumulated in the brain with the highest concentration in the hypothalamus and anterior pituitary regions, but in the cerebral cortex the concentration was very low (34). Whilst its high concentration in the anterior pituitary and hypothalamus is associated with gonadotropin release (34), the progesterone metabolite may possess an anticonvulsive potential. Actually, intravenous 5alpha-DHP reduced generalized and focal seizures in female fully kindled rats (35) and this effect was related to its interaction with GABA-A receptors (36).

Akula et al. (37) compared the anticonvulsant effect of progesterone (at doses of 20-80 mg/kg s.c). and AEDs on the intravenous pentylenetetrazol-induced seizure threshold in mice. All the studied compounds proved to have anticonvulsant dose-dependent effects. Moreover, it was shown that progesterone’s effect was stronger than that of tiagabine, GABA, adenosine, gabapentin, but weaker than that of clonazepam, diazepam, chlordiazepoxide, phenobarbital, carbamazepine, pentobarbital, pregabalin, and phenytoin (37).

Clinical data indicate that status epilepticus may be associated with a profound reduction in cerebrospinal fluid concentration of progesterone which was even 64% lower than in matched controls (38).

### Allopregnanolone

Among the known neurosteroids, ALLO is the potent and the most thoroughly examined natural endogenous positive GABA-A receptor modulator (8). This compound is responsible for the maturing of the central nervous system, and a range of behaviour in adult life, which was proved by Mòdol et al. (39). It was found that ALLO stimulated myelinisation, synaptogenesis and displayed protective and trophic properties in relation to neurons, both during the development period and in the case of disorders (40–42). Extensive research has shown that disturbances in its levels play an important role in the pathomechanism of many diseases, including neurological and psychiatric disorders (43). By modulating the level of neurosteroids in rat neonates, it was revealed that ALLO modified exploratory and anxiety-like behaviour and disrupted aversive learning in the adult animals. (39).

Lévesque et al. (44) evaluated the effect of ALLO (at doses of 9.6-12.8 mg/kg/day) on interictal jumps and high frequency oscillations (ripples: 80-200 Hz, rapid ripples: 250-500 Hz) in the pilocarpine model of mesial temporal lobe epilepsy. It was determined that the neurosteroid significantly decreased the frequency of interictal spikes and fast ripples in the hippocampal CA3 field when compared to the control group. Others showed the anticonvulsant activity of the neurosteroid which attenuated behavioral and electrographic seizures in a model of status epilepticus despite benzodiazepine resistance (45). Interestingly, hippocampal ALLO concentration was significantly lowered in rats surviving kainate-induced status epilepticus when measured 9 weeks after (46). Human data seem in line with the results observed in rodents. Meletti et al. (38, 47) analyzed ALLO concentrations in samples of cerebrospinal fluid of patients with status epilepticus. There was a significant around 30% decrease in ALLO levels. In 2017, two cases of adults with super-refractory status epilepticus were reported, in whom the introduction of treatment with ALLO (at doses of 5.6 mg/h for 5 days in the form of 120 h continuous infusion) brought positive results (48).

### Deoxycorticosterone

The next neurosteroid—DOC is a mineralocorticoid precursor whose anticonvulsant properties are connected with its enzymatic conversion to THDOC. The converted neurosteroid acts as a positive allosteric modulator of GABA-A receptors. It was found that DOC level can change under stress, because its synthesis is controlled by an adrenocorticotropic hormone. The above relationship can be explained by stress-dependent changes in the susceptibility to seizures, which are especially observed in children (49).

Anticonvulsant activity of DOC has been shown below (in the section devoted to the paediatric population). Interestingly, when evaluated in adult rats challenged with gamma-hydroxybutyric acid (a model of generalized absence seizures), THDOC potentiated seizure activity when given systemically or focally into thalamic ventrobasal nucleus (50).

### Pregnenolone Sulphate

In the case of pregnenolone sulphate, the available results point to its proconvulsive activity. Maciejak et al. (19) revealed that an increase in the level of pregnenolone sulfate resulted in increased alanine concentration—a precursor of glutamate, which contributes to the development of seizures. A similar effect was obtained by Reddy and Kulkarni (18), who studied the impact of long-term pregnenolone sulphate and DHEAS (both at a dose of 10 mg/kg/day for 4 week) administration on convulsive activity in the mouse pentylenetetrazol model. A clear cut decrease in the seizure threshold was observed. In addition, the authors drew attention to the fact that the long-term administration of DHEAS resulting in the proconvulsive activity was prevented by long-term pretreatment with progesterone (at a dose of 5 mg/kg) or ALLO (at a dose of 0.1 mg/kg) (18).

### Ganaxolone

Ganaxolone (3alpha-hydroxy-3beta-methyl-5alpha-pregnan-20-one; GNX) belongs to exogenous neurosteroids, and is the 3beta-methylated exogenous analogue of ALLO. GNX has a mechanism similar to its natural analogue, i.e. it is an allosteric modulator of GABA-A receptors (binds to both synaptic and extrasynaptic GABA-A receptors) (51, 52). In the case of the activation of synaptic GABA-A receptors, whereas the activation of extrasynaptic GABA-A receptors is connected with persistent or tonic inhibition (53). Additionally, it was confirmed that the neurosteroid did not activate classic nuclear progesterone receptors (54).

GNX displays anticonvulsive properties, which was found in many animal seizure models, including limbic seizures in the 6-Hz model, clonic seizures induced by pentylenetetrazol and bicuculline or amygdala-kindled seizures (54). Gasior et al. (55) in their experiments compared the activity of GNX with diazepam and valproate, in which they showed that GNX (effective doses of GNX predicted to protect 50% of the mice in this model [ED_50_]—3.45 mg/kg against the clonic phase), was the most effective anticonvulsant, because it decreased the convulsive activity (clonic and tonic seizures) and lethal effects of pentylenetetrazol. By comparison, diazepam displayed anticonvulsant activity involving tonic seizures and lethality, and valproate only suppressed the tonic attacks (55). These results were confirmed in behavioral and electrographic seizures in fully amygdala-kindled mice, representing a model of mesial temporal lobe epilepsy (56). Mares and Stehlíková (57) studied the activity of GNX (in 5–40 mg/kg doses) in another model—cortical epileptic afterdischarges. Rats (12, 18, and 25-day-old) were used for this study. The administration of GNX (at 40 mg/kg) inhibited progressive prolongation of cortical epileptic afterdischarges in 25-day-old rats and postponed it in 12-day-old rats. In 18-day-old rats, no significant protective effects of the drug were observed (56). GNX was also evaluated in WAG/Rij rats, a genetic model of absence epilepsy, following intracerebral injections (57). When administered bilaterally into nucleus ventralis posteromedialis, nucleus reticularis thalami, nucleus ventralis posterolateralis (thalamic nuclei), the occurrence of epileptic spike-wave discharges was worsened. However, when microinjected into the peri-oral region of the primary somatosensory cortex, GNX (at doses of 100, 200, 400 pmol and 1 nmol/side) suppressed spike-wave discharges. Practically, ALLO (at doses of 200, 400 pmol and 1 nmol/side) produced a very similar response. The effects of pregenenolone sulphate (at doses of 100, 200, 400 pmol and 1 nmol/side) were dose-dependent—the drug was generally proconvulsant at low doses and anticonvulsant at higher doses when microinjected into thalamic nuclei or somatosensory cortex (58).

GNX (at a dose of 1500 mg/day) has been also studied as an add-on therapy in randomized, placebo-controlled trials involving adult patients with partial onset epilepsy with or without secondary generalization. A Phase II trial was conducted in 147 refractory adults (100 females and 47 males in the age range of 18-69 years) (59). Out of 131 patients who completed the trial, 124 subjects were enrolled into the open-label extension study. The results of the trial were quite encouraging—GNX reduced by 18% mean weekly seizure frequency (vs a 2% enhancement in placebo group). Responder rates were evaluated as a percentage of patients in whom reduction of seizures reached at least 50%. The rates were 26 and 13% in GNX and placebo groups, respectively. A possibility that the observed beneficial results in patients on GNX could be dependent on gender or concomitant medication was excluded. Remarkably, GNX positive efficacy seems to be long-term which can be inferred from the open-label extension (59). Because of the adverse effects (mainly dizziness, fatigue and somnolence), 7% of GNX and 6% of placebo subjects had to discontinue treatment. Thirty six patients in the open-label extension continued GNX treatment for a longer period than one year and the observed adverse effects did not differ from those reported in the Phase II trial (58). Some more results from the open-label extension were published in 2013 (60). Among adverse effects observed in more than 10% of patients, were: headache (21%), convulsion (16%), fatigue (16%), fall (14%), nasopharyngitis (14%), dizziness (13%), contusion (12%), and nasal congestion (10%) (60). Detailed analysis of the results was published in 2017 (61). The authors indicate that generally, the adverse effects in the GNX group were mild to moderate. Among the untoward events leading to discontinuation in patients receiving GNX were: headache, lethargy and in placebo group: postictal psychosis, headache and convulsion. Clinical laboratory tests revealed no major disturbances—only in one patient on GNX mild thrombocytopenia was found which, however, did not result in treatment discontinuation (61).

### Minaxolone and Alphaxalone

Other exogenous neurosteroids include minaxolone (2β,3α,5α,11α)-11-(dimethylamino)-2-(ethoxy-3-hydroxypregnan-20-one), and alphaxalone (5α-pregnan-3α-ol-11,20-dione)—compounds whose mechanisms of action are also based on positive interactions at the α1 glycine receptor. However, the respective EC50 values for minaxolone or alphaxalone were roughly 10 times higher compared to GABA-A receptors (62). Both minaxolone and alphaxalone proved to be effective anticonvulsant agents against pentylenetetrazol - and bicuculline-induced convulsions in rodents. In addition, moderate anticonvulsive activity was displayed by the latter in other models, including kindling and electroconvulsions in mice (63). Also, alphaxalone exerted distinct anticonvulsant effects against NMDA-induced convulsions in mice and reduced NMDA-produced mortality (64). This protective activity was shared by other positive modulators of GABA-A receptors—ALLO and androsterone. Interestingly, the ALLO precursor, 5α-pregnane-3,20-dione, was completely ineffective in this respect. However, this neurosteroid has nothing to do with GABA-A receptors (64). Another study, however, found alphaxalone ineffective against pentylenetetrazol-induced seizure activity in mice or amygdala-kindled seizures in rats (65). Aminophylline-induced convulsions in mice were also not affected by the neurosteroid (65). Apart from the evaluation of the anticonvulsant activity of alphaxalone per se, this compound was also combined with a number of AEDs in the maximal electroshock (MES)- and pentylenetetrazol-induced convulsions in mice. First, alphaxalone (at 2.5 mg/kg) was demonstrated to raise the threshold for electronvulsions but in this effective dose it surprisingly reduced the protective activity of valproate against MES and at 2.5-5 mg/kg, alphaxalone also negatively interacted with this AED in pentylenetetrazol-induced seizures. As regards other AEDs, the anticonvulsant activity of carbamazepine, phenobarbital, phenytoin and clonazepam was not modified by the neurosteroid against MES. Similarly, the protective action of clonazepam, ethosuximide and phenobarbital remained unchanged in the presence of alphaxalone. In aminophylline-induced convulsions and amygdala-kindled seizures the protective activity of conventional AEDs (including valproate) was not affected by alphaxalone (65).

Alphaxalone was also tried in a rat model of generalized absence seizures produced by gamma-hydroxybutyric acid (50). Following its systemic or focal administration into thalamic ventrobasal nucleus, an exacerbation of seizures was noted. However, no effect was observed following its administration into thalamic reticular nucleus (50).

### Combination of Neurosteroids (Allopregnanolone and Ganaxolone) With Tiagabine or Midazolam

The examined combination of neurosteroids included ALLO and GNX with the GABA-reuptake inhibitor tiagabine or the benzodiazepine derivative - midazolam against tonic inhibition in dentate gyrus granule cells (DGGCs) or in the hippocampal kindling and 6-Hz seizure models (66). The authors provided evidence that combining individual neurosteroids with tiagabine in three standard proportions (1:1, 3:1 and 1:3) showed considerable synergism in their anticonvulsant action, and the pharmacological studies consistently pointed to the combinations’ anticonvulsant effect. Similar results were obtained in the case of neurosteroids combined with midazolam. The combination of tiagabine with GNX at a 1:1 dose ratio exerted the strongest effect. As noted by the authors, the possible mechanism behind this action may result from both the effects on extrasynaptic GABA-A receptors and tiagabine -induced increase in synaptic GABA concentration. In turn, when considering the combination of the neurosteroid with midazolam, such positive effects may be possibly related to their actions at both synaptic and extrasynaptic GABA-A receptors (66). Apart from assessing pharmacokinetic parameters (see below), Zolkowska et al. (67) also evaluated the efficacy of intramuscular ALLO and GNX (each at a dose of 3 mg/kg) in the treatment of status epilepticus induced by tetramethylenedisulfotetramine in mice. The experiments showed that both neurosteroids were effective, however, ALLO displayed slightly greater effectiveness and speed of action, which was probably connected with its greater GABA-ergic potency (67).

There are data on the pharmacokinetic properties of ALLO and GNX (67). Plasma and brain levels of ALLO and GNX were determined in naïve mice at various time points following intramuscular dosing (in both cases at a dose of 3 mg/kg). Maximum concentration values for ALLO and GNX (plasma Cmax) were 645 and 550 ng/mL, respectively. Brain levels rose more slowly and peaked at 10 min in both cases, the respective Cmax values being 845 ng/mL for ALLO and 1239 ng/mL for GNX. On the basis of all pharmacokinetic parameters, it was found that the peak brain concentrations and brain exposure (AUC) for both steroids, was approximately 3-fold the plasma exposure (additionally, GNX was shown to be higher than ALLO). In the first case, the probable cause is the slightly higher hydrophobicity of GNX than ALLO (logP values—5.423 and 5.042, respectively). In the second case, the authors indicate a higher lipophilicity of GNX as a probable cause (log P with 5.3 vs 4.9 for ALLO). Therefore, GNX initially concentrates in the brain to higher levels, being subsequently redistributed to fat tissue. Maintaining a more flatter distribution in the brain is another factor that may be responsible for the higher effectiveness of ALLO. The common feature of both neurosteroids is that both ALLO and GNX were highly bioavailable, indicating that they were almost completely absorbed following intramuscular injection (67).

## Neurosteroids and Catamenial Epilepsy

One of the possible causes of refractory epileptic seizures in women might be disturbances in the levels of progesterone and estrogen. These hormones may affect the electrical excitability of neurons and thus the seizure threshold. It was found that in the case of concentration fluctuations during the menstrual cycle, these hormones contributed to seizure exacerbation, called catamenial epilepsy. It is a dominant a type of drug-refractory epilepsy found in women of reproductive age. The fact that the characteristic feature of catamenial epilepsy is increased frequency of seizures at specific and repetitive times in the menstrual cycle serves as confirmation of how important a role is played here by the hormones (68, 69).

It was confirmed that the menstrual cycle had 3 sensitive periods in which increased seizure activity could be observed. Such increased activity usually occurs perimenstrually (C1 pattern), at ovulation (C2 pattern), and during the luteal phase (C3 pattern). The C1 and C3 phases see a drop in progesterone concentration, and the C2 period—a pre-ovulatory surge in estrogen. A decrease in progesterone concentration reduces sensitivity to the inhibitory neurotransmiter—GABA (70). It is suggested that the fluctuations in GABA-A receptor subtypes (especially in extrasynaptic δ-GABA-A receptors) could be of importance as revealed in animal experiments (71, 72). It is suspected that the occurrence of this type of seizures in female patients is usually correlated with the discontinuation of neurosteroids, hence the idea to administer exogenous progesterone during the luteal phase, which is expected to eliminate the risk of a sudden drop in the hormone levels (68). However, the results of various research efforts, including randomised studies, appear to be discordant on that matter (70).

Progesterone is synthesised in the mitochondrial membranes of body cells, and in the cerebral tissue, in several stages, the first of which is homogenous, while the remaining stages can develop in various ways, which are different in the peripheral and central compartments. There is evidence that in the peripheral compartment, the 5 beta reduction pathway predominates, in turn in the central compartment—the 5 alpha pathway which is predominant in rats, monkeys, and humans (73).

At first it was believed that progesterone itself, in its basic form, possessed anticonvulsant properties, yet subsequent studies supported a notion that its anticonvulsant activity was most likely possible through its metabolism to other compounds. In addition, it is a known fact that progesterone and its secondary metabolites ALLO and 5alpha-DHP show anticonvulsant activity (73). Furthermore, this is supported by the fact that progesterone, through its neurosteroid derivative, ALLO, increases GABA-A receptor density in the brain, thus controlling possible seizures.

Animal studies were conducted in which the progesterone metabolism was blocked by finasteride which resulted in suppressing the anticonvulsant properties of this hormone (26).

It is very likely that the progesterone nuclear receptor does not participate in the anticonvulsant mechanism which is supported by double blind, placebo controlled and randomized studies by Dan-Haeri and Richens (74), conducted in a group of patients with catamenial exacerbation. The authors examined norethisterone—a compound which is similar to progesterone but has a stronger affinity to the progesterone nuclear receptor when compared to progesterone. However, these studies did not provide the primarily expected results, as it turned out that norethisterone did not possess anticonvulsant effects (74).

There is also a case of a woman with catamenial epilepsy, involving intractable complex partial and secondary generalized seizures and accompanying hormonal disorders, in whom seizure control could not be achieved, despite the administering of various AEDs, including barbiturates, carbamazepine, phenytoin and valproate. It was progesterone therapy which brought the expected therapeutic results, but only to the point at which the dermatologist ordered the treatment with finasteride (in view of the rapidly progressive male-pattern baldness). The additional therapy resulted in the recurrence of seizures, despite continued progesterone treatment, which again might suggest it is not the hormone which has anticonvulsant properties, but its metabolites are in fact anticonvulsant (75).

Herzog et al. (76, 77) conducted a randomized, double-blind, placebo-controlled, phase III, multicenter, NIH Progesterone Trial in which they examined the efficacy of adjunctive cyclic natural progesterone therapy in a group of almost 300 women who had been diagnosed with intractable partial seizures, with or without catamenial exacerbation. The patients in the study group were given progesterone at a dose of 200 mg 3 times a day for 12 months (from the 14^th^ to the 25^th^ day of each menstrual cycle). The dose was gradually reduced in the subsequent 3 days. The experiments showed that progesterone could be a very beneficial solution for women with perimenstrually exacerbated seizures; however, in the case of women with intractable partial epilepsy, cyclic progesterone is ineffective (76, 77).

A smaller study was conducted in 36 women with catamenial epilepsy—the patients experienced seizure activity throughout the second half of the menstrual cycle, accompanied by low serum concentrations of progesterone (78). Progesterone was administered at a daily dose of 50 mg starting from the day 16^th^ and ending on day 25^th^ of each cycle. The results were encouraging. There was a 55.9% decline in the seizure frequency (primary and secondary generalized seizures) and a 63.1% decline in the partial seizure frequency—no improvement was evident in 5 patients (78).

The case is more difficult to prove with estrogen, because this hormone displays both pro- (79–81) and anticonvulsant properties (82), its mechanisms of actions being not fully understood. It is suspected that estrogen works through intracellular estrogen receptors, ER-alpha and ER-beta, which are found in nuclei of some neurons, e.g. in the hippocampus. Despite the fact that they are not very numerous when it comes to this structure, they seem to have a strong influence on the formation of synapses by neurons that do not have high levels of nuclear estrogen receptors. It was observed that non-nuclear estrogen receptors can occur outside of the cellular nuclei in dendrites, presynaptic terminals, and glial cells, where estrogen receptors can connect to second messenger systems to regulate various cellular events and signals to the nucleus through transcriptional regulators such as CREB (83). A different research group suggested that estradiol affected the hippocampal dendritic spine density through the activation of specific NMDA receptors (80). Smejkalova and Woolley (81), in turn, demonstrated that the hormone potentiated excitatory neurotransmission through a presynaptic mechanism *via* increased glutamate release. Studies are available in which the authors point to antiepileptic, and even neuroprotective, effects of estrogen. The hormone decreases neuronal death during seizures through up-regulation of the prosurvival molecule—Bcl-2, anti-oxidant potential as well as protection of NPY interneurons (82). In addition, it was demonstrated that the examined steroids caused *“the induction of dendritic spine proliferation on serotonin neurons thus thawing a profound effect on serotonergic transmission”* by the activation of 5-HT3 and 5-HT1A receptors (82).

It is possible that androgens also display a bimodal character. Animal studies have shown that testosterone can cause convulsive episodes to be aggravated, but, on the other hand, it was demonstrated to possess anticonvulsant properties which was connected with its transformation to various metabolites. The first case is a testosterone metabolism to 5α-DHT by 5α-reductase, which is then reduced by 3α-hydroxysteroid oxidoreductase enzyme resulting in the synthesis of anticonvulsant metabolite 3α-androstanediol, a potent GABA-A receptor modulating neurosteroid (84). In the second case, reduction of testosterone by aromatase generates proconvulsant 17-β estradiol (84, 85). Tutka et al. (86), in their experiments, assessed the effects of androsterone on the anticonvulsant properties of AEDs against MES-induced convulsions in mice. It was shown that androsterone, when administered alone (80 mg/kg), elevated the seizure threshold. This was not observed at lower doses (5-40 mg/kg). When combined with AEDs, androsterone (at 40 mg/kg) significantly enhanced the anticonvulsant activity of phenobarbital, gabapentin and carbamazepine, but it did not affect the protective activity of phenytoin, lamotrigine, oxcarbazepine, topiramate, or valproate. The observed lack of androsterone’s effect on the brain total concentration of AEDs suggests that the positive effect of this neurosteroid was not connected with pharmacokinetic interactions (86). Effects of neurosteroids on seizure susceptibility have been summarized in **Table 1**.

**Table 1:** Neurosteroids—mechanisms of action and effects on seizure activity.

	Neurosteroid	Mechanism of neurosteroid action	Anticonvulsant action	Proconvulsant action	Experiments on animal models
**Endogenous neurosteroids**	Allopregnanolone	positive allosteric modulator of GABA-A receptors (38)	+	−	Kainite, PTZ, 4-aminopyridine model (13)
	Androsterone	positive allosteric modulator of GABA-A receptors	+	−	MES model (86)
	Deoxycorticosterone	positive allosteric modulator of GABA-A receptors (46)	+ −	- +	MES and PTZ model in juvenile rats (87, 88) hippocampal kindling in juvenile rats (88) Model of generalized absence seizures in rats (50)
	Dehydroepiandrosterone sulfate	negative modulator of GABA-A receptors (18) modulates the NMDA receptor (19)	−	+	PTZ model (18)
	Pregnenolone sulfate	negative modulator of GABA-A receptors (18) modulates the NMDA receptor (19)	−	+	seizures induced by picrotoxin, bicuculline and NMDA (19)
	Progesterone	positive allosteric modulator of GABA-A receptors (59)	+	−	Amygdala kindling model in rats (19, 29), hippocampal kindling model (30), WAG/Rij rats, the genetic absence model (31) kainate model in rats (32)
**Exogenous neurosteroids**	Alphaxalone	positive allosteric modulator of GABA-A and α1 glycine receptor (59)	+ −	− +	PTZ- and bicuculline-induced convulsion (63) Model of generalized absence seizures in rats (50)
	Ganaxolone	positive allosteric modulator of GABA-A receptors (47, 48)	+	−	6 Hz model, PTZ, bicuculline seizures, amygdala-kindled seizures (54, 55). cortical epileptic afterdischarges in rats (57)
	Minaxolone	positive allosteric modulator of GABA-A, α1 glycine receptor (59)	+	−	PTZ- and bicuculline-induced convulsions (62)

Experiments were carried out in mice unless stated otherwise. MES, maximal electroshock; NMDA, N-methyl-d-aspartate; PTZ, pentylenetetrazol; +, present; −, absent.

## Neurosteroids and Seizure Activity In The Pediatric Population (Human And Animal Data)

Epilepsy in children is a crucial problem, because its prevalence is greater than epilepsy in the adult population. This is associated with the increased susceptibility of immature cerebral tissue to spontaneous neuronal discharges. Due to the high risk of irreversible pathological changes, it is recommended to commence treatment as soon as possible. In addition, it has been shown that not only is an early start important, but also the appropriate type of treatment administered. The authors have proved that the introduction of the wrong AED therapy in the first line of treatment has long-term effects, including the reduction of the effectiveness of subsequent treatment courses with the appropriately selected AEDs (89). The treatment of neonates has proved to be extremely challenging, because epileptic seizures occurring in this group of patients are usually refractory to standard pharmacological treatment. There are cases in which AED administration can even aggravate the seizures (90). Consequently, there is a huge need to introduce new therapies which can be used in the youngest epilepsy patients.

Although ALLO possesses anticonvulsant properties, it is uncertain what effects it would display in children with epilepsy. With paediatric patients it should be borne in mind that GABA may become a depolarizing neurotransmitter in the brain, and GABAergic inhibition can be a result of both membrane hyperpolarization and a stimulus. In such a case GABA can act as both a stimulatory and an inhibitory neurotransmitter in an immature brain (91, 92).

The stimulating nature of GABAergic neurotransmission in the neonatal period can stem from, on the one hand, an increase in the level of Na^+^–K^+^–2Cl^−^ co-transporter (importing Cl^−^ inside the cell—NKCC1), and, on the other, a decrease in the level of K^+^–Cl^−^ co-transporter 2 (mediating Cl^−^ transport out of the cell, KCC2). As a result, a high level of chloride ions is noted inside a cell (93). The researchers are of opinion that the activity of NKCC1 was high in the hippocampal and cortical neurons, especially in the first week of life, in both rats and humans, and it gradually decreased with time which was particularly apparent from the 14th day of life (94, 95). It is believed that this is connected with the depolarizing to hyperpolarizing shift of GABA receptors, which starts around the 8-10th day of life and ends on the 14th day. This was proven in experiments on rat CA3 hippocampal pyramidal cells (94, 96). Kolbaev et al. (97) in their *in vivo* studies on the CA3 region of hippocampal slices from immature (postnatal day 4-7) rats, showed that synaptic GABAergic neurotransmission suppressed epileptic discharges, and, in turn, strengthened extrasynaptic GABAergic drive caused epileptiform activity. It is also important to note that δ-subunit expressing GABA-A receptors which participates in extrasynaptic GABAergic transmission, can be a potential target of neurosteroids, which was confirmed in a mouse model (98). Therefore, it is important to study the effects of ALLO on neuronal excitability in an immature brain. There are studies which show the anticonvulsant effect of ALLO in an immature brain. Sharopov et al. (99) examined the impact of ALLO on epileptiform activity in an in-toto hippocampus preparation of early postnatal mice (postnatal days 4-7). It was found that the neurosteroid, through a positive modulation of GABA-A receptors, did not show any impact on ictal-like epileptiform activity, however, an increase in interictal epileptiform events was observed. Additionally, based on studies using a patch-clamp, it was determined that ALLO prolonged the decay of GABAergic postsynaptic currents, but did not result in changes in the case of tonic GABAergic currents, which could result in an increase in the neuronal excitability of an immature brain (99).On the other hand, in the case of *in vivo* studies, results by Dhir and Chopra (13) should be taken into consideration. They examined the effects of a ALLO in 9-day-old rat neonates against seizures induced by kainic acid, pentylenetetrazol or 4-aminopyridine. Treatment with ALLO (5 and 10 mg/kg) delayed the occurrence of status epilepticus, but did not impact on the myoclonic jerks or the mortality rate in the kainic acid group. In the case of convulsions evoked by 4-aminopyridine, neurosteroid treatment—only at a higher dose of 10 mg/kg—contributed only to a delay in seizure activity. ALLO treatment (at 5 mg/kg) in the pentylenetetrazol group, resulted in the protection of rat neonates against seizures and death (13). Another research group evaluated the impact of neurosteroids (ALLO—at doses of 20, 30 and 40 mg/kg, pregnanolone and triethylammonium 3alpha-hydroxy-20-oxo-5alpha-pregnan-21-yl hydrogensuccinate (THDOC-conjugate—at 20 and 40 mg/kg) on rat neonates in 3 age groups: 12-, 18- and 25-day-old rats (in the case of ALLO treatment) and in two age groups: 12- and 25-day-old rats (in the case of other neurosteroids). Convulsions were induced through electrical stimulation using intracerebral electrodes. The experiments showed that all neurosteroids displayed anticonvulsant properties (the strongest effect was observed with pregnanolone) in 12-day-old rats, but only a tendency in 25-day-old ones. In turn, in the case of ALLO, no such tendency was observed in 18-day-old rats (100). Subsequent studies also assessed the effects of ALLO (at doses of 5 – 40 mg/kg) on convulsive activity in the pentylenetetrazol model, but this time the treatment involved 7-, 12-, 18-, 25- or 90-day-old rats (100). Similarly to the previous experiments, the neurosteroid supressed generalized tonic-clonic and minimal clonic seizures, in particular in the 12-day-old rat study group. The weakest anticonvulsant effect was, in turn, observed in the 90-day-old rat study group. The above results can be explained by the fact that the effects of ALLO lasted longer in young, than in adult rats (101). Other studies, also conducted by the same research team, evaluated the anticonvulsant properties of an analogue of ALLO—3alpha-hydroxy-21xi,22-oxido-21-homo-5alpha-pregnan-20-on (at 40 mg/kg) and GNX (at 60 mg/kg) in a group of 12- and 25-day-old rat neonates against pentylenetetrazol-induced convulsions. A similar activity by both neurosteroids was found, but a more-favourable result was observed in younger rats (102). Based on the above studies it can be concluded that neurosteroids are the most effective when used in 12-day-old rat neonates, which the authors explain by the increased sensitivity towards the anticonvulsant potential of neurosteroids in younger rats (100). It is worth noting that, despite the positive effects of neurosteroids in rat neonates, one-week-old animals were excluded from the above studies while their brains roughly resemble those of pre-term children (103). In the case of preterms, it is worth stressing that they are particularly prone to CNS disorders. The high concentration of neurosteroids in advanced pregnancy protects the fetal brain against hypoxia and supports the normal development of CNS. When the ALLO level decreases, it brings about overexcitability in the neurons and increases the risk of brain damage secondary to hypoxia. Following birth (both normal and preterm) the neurosteroid level is found to be lower, which is particularly unfavourable for a preterm (104).

Age-dependent effect was also evident for DOC which exhibited clear cut anticonvulsant effects in neonatal, infant, weanling and juvenile rats against PTZ-induced convulsions (87). DOC at low dose (10 mg/kg) lost its protective effect and its anticonvulsant activity after a high sedating dose (40 mg/kg) was significantly reduced after puberty (87). Further experiments provided evidence that this neurosteroid was effective against MES (87, 88) and hippocampal kindling in 15-day-old rats, too. Much higher doses of DOC were required for adult rodents (88). Interestingly, the anticonvulsant efficacy of DOC against PTZ was significantly reduced by finasteride, indicating that the neurosteroid acts *via* its metabolites, dehydrodeoxycorticosterone and THDOC (105). Actually, these metabolites were found effective against PTZ-induced convulsions in infant (15-day-old) rats (105).

Kaciński et al. (43) assessed the effects of ALLO on pseudoseizures in children. The children were divided into 3 groups: (I) children with pseudoseizures without treatment with AEDs; (II) children with pseudoseizures and treated with AEDs; (III) children without pseudoseizure attacks and no treatment with AEDs. The results showed no significant changes in ALLO levels, both before and after provoking pseudoseizures by placebo. It might point to the fact that during pseudoseizure attacks, in contrast to epileptic seizures, endogenous anticonvulsant and anxiolytic neurosteroid levels do not increase. In addition, the authors noted that the low ALLO level can intensify the stress response and contribute to the occurrence of pseudoseizures (43). Broomall et al. (106) were the first to describe two children with super-refractory status epilepticus. This disorder is characterized by resistance to benzodiazepine and barbiturate treatment, which is most probably connected to the internalization of synaptic GABA-A receptors. ALLO was used, and it made it possible to discontinue general anesthetic infusions (106). In their studies, Grosso et al. (107), suggest that circulating ALLO significantly increases in the post-ictal phase. They found no significant differences in the post ictal serum ALLO between patients with partial seizures and those with generalized seizures. They included three groups of subjects in the study. Group 1 consisted of 18 children affected by complex partial seizures. Group 2 consisted of 11 children presenting with generalized epilepsy. Group 3 consisted of 20 healthy age-matched subjects. Serum ALLO levels were assayed in the inter-ictal phase and within 30 min after an epileptic event (107). A possibility exists that a reduced blood ALLO concentration may be causally related to pathophysiology of protocadherin 19 female limited epilepsy (PCDH19-FE) which is actually a clear cut infantile onset syndrome with autism and intellectual disability in some cases. Genes, encoding enzymes involved in the metabolism of steroid hormones, were evaluated in transcriptomes of primary skin fibroblasts. Out of the AKR1C1-3 genes, significant changes were found in AKR1C3 in terms of reduced mRNA and protein concentrations in PCDH19-FE patients (108). Obviously, the reduced blood concentration of ALLO followed in these patients which could be responsible for the development of PCDH19-FE (108). Further studies on PCDH19-FE patients confirmed the earlier findings of Tan et al. (108), showing that the serum concentration of ALLO was significantly reduced not only in baseline but after stimulation with ACTH as well (109). Strikingly, the synthesis of pregnenolone sulphate was even more reduced than that of ALLO so a hypothesis that seizures could be generated by an assumed imbalance in the ALLO/pregnenolone sulphate ratio proved unlikely (109).

One of the first studies to evaluate the effect of GNX on convulsive activity in the paediatric population was conducted approximately 20 years ago. Kerrigan et al. (110) examined this neurosteroid in the population of children with refractory infantile spasms, or with continuing seizures following a prior history of infantile spasms (aged 7 months to 7 years). It was determined that GNX reduced the number and frequency of seizures from 25% to > 50%. In turn, Pieribone et al. (111) evaluated the anticonvulsive effects of GNX in children (aged 5-15) with highly refractory focal and generalized crypto-symptomatic epilepsy. The results confirmed that the neurosteroid produced anticonvulsive effects. In some patients, adverse events were observed; however, all of them were described as mild to moderate. Yawno et al. (112) suggested that GNX should also be administered in the case of convulsions in neonates and preterms. The author draws attention to the fact that the drug, on one hand, is very safe, because most probably it produces no negative effects on the neonates’ brains, and, on the other, it can prevent or considerably reduce the prevalence of permanent damage resulting from hypoxia, preterm birth, or epilepsy (112). Clinical research has been presented in **Table 2**.

**Table 2:** Neurosteroids—clinical research.

Neurosteroids	Type of seizures	Trial group	Doses applied	References
**Allopregnanolone**	generalized convulsions and myoclonus	Adults	670.8 mg (5.6 mg/h for 5 days), intravenous solution, containing 6% hydroxypropyl‐β‐cyclodextrin in 0.9% sodium chloride injection	(48)
	super-refractory status epilepticus	Children	iv solution (0.5 mg/ml in 0.9% NaCl with 6% Captisol for 5 days	(106)
	complex partial seizures generalized epilepsy	Children (aged 11 months to 7.8 years)	no data	(107)
**Progesterone**	catamenial epilepsy	Adults (women)	200 mg three times daily on days 14–25, followed by a 3-day taper) of each cycle	(75)
	intractable partial seizures, with or without catamenial exacerbation.	Adults (women)	200 mg 3 times a day for 12 months (from the 14th to the 25th day of each menstrual cycle)	(76, 79)
	catamenial epilepsy	Adults (women)	50 mg starting from the day 16th and ending on day 25th of each cycle.	(78)
**Ganaxolone**	partial onset epilepsy with or without secondary generalization	Adults (aged 18-69 years)	1,500 mg/day	(59)
	refractory infantile spasms, or with continuing seizures following a prior history of infantile spasms	Children (aged 7 months to 7 years).	the dose of ganaxolone was progressively increased to 36 mg/kg/d (or to the maximum tolerable dose) over a period of 4 weeks and then maintained for 8 weeks prior to tapering and discontinuation of the attack	(110)
	highly refractory focal and generalized crypto-symptomatic epilepsy	Children (aged 5-15 years)	ganaxolone in a 1:1 complex with β‐cyclodextrin in a dose escalation (1 mg/kg, b.i.d. to 12 mg/kg t.i.d.) schedule over 16 days	(111)

## Conclusions

It is evident that endogenous and exogenous neurosteroids may exert anti- or proconvulsant activity. As already indicated above, the anticonvulsant activity is associated with the positive modulation of GABA-A receptors and by the way, some anticonvulsant neurosteroids were documented to inhibit aspartate release from rat hippocampal slices (64). The anticonvulsant neurosteroids comprise for instance: androsterone, progesterone, ALLO, alphaxalone, GNX. In contrast, proconvulsant neurosteroids (pregnenolone sulfate or DHEAS) negatively modulate the function of GABA-A receptor complex. Modulation of seizure activity may be also associated with sigma receptors as a sigma receptor antagonist, rimcazole, lowered the convulsive threshold on one hand, but on the other, it potentiated the anticonvulsive activity of phenobarbital and valproate against MES in mice (113). When considering anticonvulsant effects of neurosteroids, progesterone block of voltage-operated calcium channels may be of importance as many agents expressing this mechanism of action express anticonvulsant activity (114). Modulation of seizures *via* glycine 1 receptors seems rather unlikely *in vivo* due to the relatively weak binding of neurosteroids to these receptors. A completely different situation may be encountered in absence seizures as revealed from WAG/Rij rats. The observed worsening of absence seizures in 6-month-old WAG/Rij rats may be associated with the up-regulation of thalamic α-4 and δ GABA-A receptor subunits which probably leads to an enhanced GABA-ergic inhibition of thalamic relay neurons (115).

Because intractable seizures generally require adjuvant treatments, interactions of neurosteroids with AEDs are of particular importance. Preclinical data point to the beneficial effects of androsterone when combined with carbamazepine, gabapentin and phenobarbital (86). Also, combinations of ALLO and GNX with tiagabine or midazolam were found highly effective against hippocampal kindling and 6 Hz-induced convulsions in mice (66). The results concerning alphaxalone are not that encouraging as this neurosteroid diminished the anticonvulsant activity of valproate against MES or pentylenetetrazol in mice. Unexpectedly, the total brain concentration of valproate was elevated by alphaxalone (65).

Clinical data are generally positive, pointing to progesterone as an effective drug against catamenial epilepsy (76–78). Nevertheless, the hormone is ineffective as regards intractable partial seizures in women (76). A very promising AED, GNX, has entered phase III study (116). It has shown a considerable efficacy as an adjuvant against infantile spasms (110), highly refractory epilepsy in children (111) and possibly ganaxolone will prove effective for the management of neonatal seizures following hypoxic injury (112). Positive results are also available from a trial conducted on adult patients with partial epilepsy (59–61).

Using neurosteroids in the paediatric population deserves special attention, especially in the period of the last semester of gestation up till the first several years after birth. This is actually the period of the intensive synaptogenesis (117). Experimental data obtained from immature animals clearly indicate the drugs enhancing GABA-mediated inhibition can induce massive neuronal apoptosis similarly to alcohol which is known to cause fetal alcohol syndrome (117).Whether neurosteroids *via* GABA-mediated events may cause remote clinical problems in the paediatric population due to the enhanced apoptosis is at present not known.

## Author Contributions

BM was involved in writing most parts of the first draft and designing the table and schemes. MC-K dealt with some aspects of the treatment of pediatric patients with neurosteroids. SC prepared conclusions and critically read the whole manuscript, performing necessary additions (with relevant references) and revisions.

## Funding

This study received no external funding. The authors greatly acknowledge a statutory grant (DS 475/20) from Medical University of Lublin. Medical University of Lublin had no role in study design, data collection and analysis, decision to publish, or preparation of the manuscript.

## Conflict of Interest

SC was granted financial support from Bayer, GlaxoSmithKline, Janssen, Novartis, Sanofi-Aventis for lecturing (until 2014). He also received an unrestricted grant from GlaxoSmithKline for the period of 2006-2007.

The remaining authors declare that the research was conducted in the absence of any commercial or financial relationships that could be construed as a potential conflict of interest.
